# Musical Performance in Adolescents with ADHD, ADD and Dyslexia—Behavioral and Neurophysiological Aspects

**DOI:** 10.3390/brainsci12020127

**Published:** 2022-01-18

**Authors:** Christine Groß, Bettina L. Serrallach, Eva Möhler, Jachin E. Pousson, Peter Schneider, Markus Christiner, Valdis Bernhofs

**Affiliations:** 1 Jazeps Vitols Latvian Academy of Music, K. Barona Street 1, LV-1050 Riga, Latvia; Christine.michaela.gross@jvlma.lv (C.G.); jachin.edward.pousson@jvlma.lv (J.E.P.); p.schneider.hd@web.de (P.S.); valdis.bernhofs@jvlma.lv (V.B.); 2 Department of Neuroradiology and Section of Biomagnetism, University of Heidelberg Medical School, University of Heidelberg, INF 400, 69120 Heidelberg, Germany; bettinaserrallach@icloud.com; 3 Department of Child and Adolescent Psychiatry, Saarland University Hospital, G-66421 Homburg, Germany; eva.moehler@uks.eu; 4 Centre for Systematic Musicology, Faculty of Arts and Humanities, University of Graz, Glacisstraße 27, A-8010 Graz, Austria

**Keywords:** musical performance, rhythm, pitch, neurodevelopment disorders, auditory cortex, auditory-evoked fields, magnetencephalography

## Abstract

Research has shown that dyslexia and attention deficit (hyperactivity) disorder (AD(H)D) are characterized by specific neuroanatomical and neurofunctional differences in the auditory cortex. These neurofunctional characteristics in children with ADHD, ADD and dyslexia are linked to distinct differences in music perception. Group-specific differences in the musical performance of patients with ADHD, ADD and dyslexia have not been investigated in detail so far. We investigated the musical performance and neurophysiological correlates of 21 adolescents with dyslexia, 19 with ADHD, 28 with ADD and 28 age-matched, unaffected controls using a music performance assessment scale and magnetoencephalography (MEG). Musical experts independently assessed pitch and rhythmic accuracy, intonation, improvisation skills and musical expression. Compared to dyslexic adolescents, controls as well as adolescents with ADHD and ADD performed better in rhythmic reproduction, rhythmic improvisation and musical expression. Controls were significantly better in rhythmic reproduction than adolescents with ADD and scored higher in rhythmic and pitch improvisation than adolescents with ADHD. Adolescents with ADD and controls scored better in pitch reproduction than dyslexic adolescents. In pitch improvisation, the ADD group performed better than the ADHD group, and controls scored better than dyslexic adolescents. Discriminant analysis revealed that rhythmic improvisation and musical expression discriminate the dyslexic group from controls and adolescents with ADHD and ADD. A second discriminant analysis based on MEG variables showed that absolute P1 latency asynchrony |R-L| distinguishes the control group from the disorder groups best, while P1 and N1 latencies averaged across hemispheres separate the control, ADD and ADHD groups from the dyslexic group. Furthermore, rhythmic improvisation was negatively correlated with auditory-evoked P1 and N1 latencies, pointing in the following direction: the earlier the P1 and N1 latencies (mean), the better the rhythmic improvisation. These findings provide novel insight into the differences between music processing and performance in adolescents with and without neurodevelopmental disorders. A better understanding of these differences may help to develop tailored preventions or therapeutic interventions.

## 1. Introduction

Musical performance is a very complex human capability and requires a broad variety of skills, including precise instrument/vocal control and technique with accuracy of notes, rhythm and phrasing as well as interpretational skills such as appropriate tempo and dynamic, suitable sense of style and involvement in the music [1]. 

For the assessment of musical ability, several well-designed musical perceptual measurements such as the Seashore test [2], the Intermediate Measures of Musical Audiation [3], the Advanced Measures of Musical Audiation [4], the Montreal Battery of Evaluation of Amusia [5] and the more recently developed Profile of Music Perception Skills [6] are available. In addition, there are self-report questionnaire inventories such as the Goldsmiths Musical Sophistication Index (Gold-MSI), which measures musical sophistication and consists of five factors: active engagement, perceptual abilities, musical training, singing ability and emotions [7]. However, to date, there are only few musical performance measures which focus either on the reproduction of rhythmical and melodic sequences [8] or on performing familiar or unfamiliar songs [9,10].

Until now, research mainly concentrated on perceptual musical ability tests. Music perception measures mainly use objective measures with correct or incorrect assessment options. As an acoustic analysis of music performance relies on accuracy, it has the advantage that findings are reproducible [11,12]. However, computerized methods can reach their limits. For instance, when individuals play musical pieces or perform songs technically perfect but in an inaccurate pitch, they could be evaluated poorly, even though the performance may be quite good [10,13]. In contrast, music performance assessments are often based on very time-consuming approaches [14] with rating scales based on certain criteria chosen by experts in the field [15,16,17,18,19]. These rating scales may be used in flexible ways and can therefore be adapted according to specific rating criteria [11], which has also the advantage that longer sequences can be assessed [14]. Even though rating scales are subjective, research found a high correlation between acoustic and subjective measures of musical performance [11]. The increased reliability of measures based on rating scales can be achieved by using more than one rater—an approach we decided to use in this investigation and which has been applied previously [9,17,18,20]. As our cohort included individuals with dyslexia, ADD and ADHD, we decided to use rating scales, since piloting has shown that individuals with diagnoses more frequently sang parts of the musical pieces out of tune.

So far, there are contradictory findings regarding the link between music perception and performance [21,22]. Some researchers noted a relationship between music perception and production [23,24], while others could not detect a relationship between both [25]. This dissociation between the perception and production of musical stimuli gained increasing interest in impairment studies. These studies assume that if one capacity is impaired, the other could possibly be spared. In this respect, alternative explanations for deficits were put forward. For instance, while it is generally accepted that amusics’ poor singing ability stems from poor pitch perception deficits, recent research found evidence that amusics’ poor singing ability can be explained by the inability to control sensorimotor translations [26,27]. Conduction aphasia, which leads to spontaneous speech production impairment, is understood as a deficit in sensory–motor integration [28], and stuttering improves alongside gaining sensory–motor control of the vocal motor apparatus [29]. Sensorimotor synchronization is a crucial aspect of referential behavior and describes the rhythmic coordination of perception and action, which is also a fundamental aspect required in musical activities [30]. Sensorimotor synchronization is a crucial aspect of referential behavior and describes the rhythmic coordination of perception and action, which is also a fundamental aspect required in musical activities [30]. So far, sensorimotor skills have mainly been assessed by the finger tapping paradigm, which measures the synchrony between the tapping of the index finger and the pacing stimuli [31]. More recently developed test batteries, such as the *Assessment of Auditory Sensorimotor and Timing Abilities (BAASTA)* [31] and the *Harvard Beat Assessment Test* (H-BAT) [32], provide information about perceptual and sensorimotor timing ability. There is evidence that musical training enhances sensorimotor synchronization [32], and musicians show more elaborate synchronization skills, lower tapping variability and greater perceptual sensitivity compared to controls [33].

As musical performance requires the integration of multimodal sensory and motor information, professional musicians not only demonstrate enlargements in the motor cortex but also neuroplastic changes at a cellular level [34]. The auditory cortex is widely linked to various brain regions, including prefrontal and parietal regions, and is involved in complex auditory and non-auditory functions, such as spectral and holistic listening modes [35], absolute and relative pitch [36,37], sensorimotor [38,39,40], cognitive [41] and language-related [42,43,44] functions. 

Neurophysiological research suggests that the neural processing of language and music may be shared since acoustic signals of speech show similarities to music in temporal and spectral complexity [45,46]. This may be one fundamental reason why individuals with diagnosed neurodevelopmental disorders show deficits in both music and language processing [47,48]. Models such as the “OPERA” hypothesis postulate that benefits in speech processing induced by musical training are based on five conditions: overlap, precision, emotion, repetition and attention [49]. The OPERA hypothesis mainly focusses on perceptual parameters. Other models such as the Precise Auditory Timing Hypothesis (PATH) suggest that auditory–motor entrainment and phonological awareness both depend on the same mechanisms: neural timing and its integration into motor and cognitive networks [50]. Therefore, it can be postulated that musical training with emphasis on entrainment also trains phonological skills [50]. The *Processing Rhythm in Speech and Music* (PRISM) framework defines precise auditory timing, the synchronization/entrainment of neural oscillations to external rhythmic stimuli and sensorimotor coupling as the three common mechanisms which underly music and speech rhythm processing [47]. The PRISM model has not only been introduced to show overlaps between music and speech perception and production but also provides a framework for developmental speech disorders. This framework unites auditory processing, crucial for the detection of timing deviations, the synchronization and entrainment of neural oscillations and sensorimotor coupling, which links perception to production [47].

There is a growing body of evidence that the anatomy and function of the auditory cortex is altered in neurodevelopmental disorders such as dyslexia, attention deficit hyperactivity disorder (ADHD) and attention deficit disorder without hyperactivity (ADD) [51,52,53,54,55]. Dyslexia and AD(H)D belong to the most common neurodevelopmental disorders in children and adolescents, with a worldwide prevalence of about 5–10% [56,57], and show a high level of comorbidity [58,59,60]. Dyslexia is a specific learning disability characterized by difficulties with accurate and/or fluent word recognition and by poor spelling and decoding abilities. A poor discrimination of basic sound features and sequential acoustic patterns may lead to suboptimal speech representation, constraining the development of phonological representations [61] and reading and spelling skills [62]. Individuals with dyslexia not only have timing difficulties in language and music perception, performance and lack motor control [63,64,65,66] but also a large variety of auditory deficits, ranging from basic to more complex auditory processing deficits [51,62,65,67,68,69]. In addition, they have impairments in higher-order cognitive processing (e.g., executive functions) and cognitive skills (e.g., cognitive flexibility) [70,71,72]. Children with developmental disorders have been found to exhibit underlying timing deficits which were not only seen as predictors for the disorders [70,72] but also as triggers [67].

AD(H)D is characterized by the key symptoms of hyperactivity, impulsivity and/or inattention. According to the International Statistical Classification of Diseases German Modification [73], two subtypes (namely ADHD and ADD) are distinguished. Patients affected by AD(H)D show broad deficits including motor deficits, sensorimotor integration impairments, perceptual timing deficits, temporal foresight and rhythm-related deficits such as the poor differentiation of temporal auditory parameters and the desynchronization of temporal patterns [74,75,76,77,78,79,80,81,82]. Moreover, difficulties in hearing and understanding oral instructions [83] and a lack of the ability to move to a beat and detect deviations from a beat [84] can be found.

There is scarce literature dealing with characterizing features of musical ability in AD(H)D subtypes/ presentations. Noreika and colleagues demonstrated that perceptual timing and temporal foresight is less impaired in ADD than in ADHD [77]. Children with ADHD show higher-order auditory processing deficits, including impairments in perception of rhythm and melody, and children with ADD demonstrate no auditory impairment at all [51].

In previous studies, we observed auditory neurofunctional anomalies in children with dyslexia, ADHD and ADD. While group-averaged P1 source waveform responses were well-balanced in controls, the disorder groups showed a pronounced P1-asynchrony [51,53]. While dyslexics showed impairments in elementary (e.g., frequency, tone onset and duration) and complex auditory sound discrimination (meter, rhythm, melody, harmonic sound perception and phoneme discrimination), children with ADHD only performed worse in sequential auditory pattern recognition. In contrast, there were no auditory deficits in children with ADD. Musical training in children with dyslexia, ADHD and ADD lead to a markedly diminished asynchrony of the primary auditory answers [51].

To our best knowledge, to date, there is no research focusing on musical performance in neurodevelopmental disorders such as ADHD, ADD and dyslexia. Therefore, we wanted to close this research gap.

Hence, the goal of this study was to (a) evaluate the group-specific characteristics of musical performance in adolescents with dyslexia, ADHD and ADD, and (b) to investigate whether the potentially found differences in performance can be correlated to the response pattern of the auditory cortex as measured using magnetoencephalography (MEG).

Due to the abovementioned auditory impairments, we hypothesized that the disorder group would perform worse in the musical performance assessment scale than the control group. Within the disorder group, we assumed that adolescents with ADD would perform better in the musical performance than adolescents with ADHD or dyslexia, since the latter both showed auditory impairments in previous research. Additionally, we wanted to uncover whether our groups could also be differentiated based on the response pattern latencies of the auditory cortex as measured using magnetoencephalography (MEG). Based on the statistical analysis, we wanted to analyze whether the musical performance and MEG variables which discriminate our groups best are also correlated with each other.

## 2. Materials and Methods

### 2.1. Participants

A total of 96 adolescents participated in this study. The subjects were 19 adolescents with ADHD (2 females; 17 males; M = 14.05, SD = 1.43), 28 with ADD (8 females; 20 males; M = 14.32, SD = 1.78), 21 with dyslexia (10 females; 11 males; M = 13.64, SD = 1.17) and 28 unaffected controls (14 females; 14 males; M = 14.48, SD = 1.12) (see Table 1).

All adolescents in this investigation were part of the larger combined cross-sectional and longitudinal research project “AMseL” (Audio- and Neuroplasticity of Musical Learning) addressing the effects of musical practice on the brain and cognition from the primary school age to young adulthood. The AMseL project was supported by the German Federal Ministry of Education and Research (BMBF) and the Germany research foundation (DFG), conducted at the University of Heidelberg (2009–2020), and partially accompanied by the cultural education program “An Instrument for Every Child (JeKi)”. For this study, participants with dyslexia, ADHD and ADD with musical expertise were recruited from all over Germany and Switzerland.

Affected participants were diagnosed by a child psychiatrist, and a written diagnosis was obtained. Subjects who received the classifications F90.0/F90.1 (ADHD) or F98.80 (ADD) according to the International Statistical Classification of Diseases and Related Health Problems German Modification, 10th Revision (ICD-10-GM) were included in the study. Dyslexics were diagnosed according to the Pediatric Neurology standards of the University Hospital Heidelberg, using ELFE [85] for reading comprehension, HSP1–10 [86] to assess spelling skills and H-LAD to assess phoneme discrimination [87]. All participants had normal hearing (defined as ≤20 dB HL pure-tone thresholds from 250 to 8000 Hz) and no known comorbidities or history of neurological disorders. As is known from previous studies, the proportion of males was higher in the ADHD and ADD group [51,88,89]. The musical performance and neurophysiological correlates were measured by means of the Music Performance Assessment Scale (MuPAS) and MEG in a cross-sectional design.

This study was approved by the responsible ethical committee. Parents provided informed consent, and adolescents provided informed assent.

### 2.2. Musical Background

As published in previous studies, in order to assess the musical practice of participants, a cumulative musical practice index (musical status) was calculated by combining participants’ statements regarding the number of years of formal music education received and the amount of time spent practicing [51,53]. A one-way ANOVA test confirmed that there was no significant main effect of the cumulative musical practice index and the disorder groups, F(3, 94) = 1.61, *p =* 0.192, ω = 0.18. Additionally, the number of musical instruments played (including singing) were reported. Overall, 8 subjects played four instruments, 8 played three instruments, 30 played two instruments, 38 played one instrument and 12 used to play at least one instrument but did not practice any instrument at the moment of measurement. Additionally, 45 participants played or sang in an ensemble (e.g., choir, orchestra or brass ensemble).

### 2.3. Musical Performance Measurement: Music Performance Assessment Scale (MuPAS)

Musical performance has been defined by a number of diverse musical capacities. These include sight-reading (performing unfamiliar music from notation), performing well-prepared pieces from memory or from notation, improvising, playing by ear (performing music from aural presentation) and singing familiar and unfamiliar melodies [1,9,10,90,91,92]. Musical performance depends on accurate timing ability [93], metrical structure and on the organization of a piece of music [94,95]. The accuracy of pitch and intonation is of major importance to maintain the harmonicity and the aesthetic quality of a musical performance [96]. Although most musical measurements used in the scientific context differ as to the underlying concept of musicality [97], rhythm and pitch are two of the main overarching dimensions of music [98]. In this context and based on previously used musical production tasks [99], we developed a musical performance measurement, the Music Performance Assessment Scale (MuPAS), which focuses on two dimensions: pitch accuracy and rhythmic ability.

The MuPAS measures the ability and competence to perform music by singing or playing a musical instrument in relation to the musical experience. It consists of 2 modules—rhythm and pitch—with 4 tasks for each module. All instructions were provided by a recorded female voice. For the rhythmic tasks (RTs), the participants were instructed to:

- Listen to a rhythmic phrase twice (2/4, 6 bars including on the beat rhythmic accents and slight changes in dynamics) and repeat it by clapping as precisely as possible without any time limit after the second listen (RT1);
- Listen to 3 short rhythmic phrases twice (2/4, 2 bars each including simple and compound division) with a metronome clicking in the background, and then rhythmically improvise to each phrase by handclapping during the second listen while keeping the meter (RT2);
- Sight read a rhythmic phrase by handclapping, which was presented on a single line staff including quarters, semi-quarters and dotted notes (RT3);
- Memorize 3 subsequent rhythmic phrases with free chosen titles (valley—pattern characterized by flat voicing, mountain—pattern characterized by partly ascending voicing and cliff—pattern characterized by partly descending voicing) and rename two randomly played phrases (RT4).

Melodic tasks (MTs) were similar to the RTs in their main structures and provided the following instructions:

- Memorize 3 music phrases with free chosen titles (forest—pattern characterized by chord progression, river—–pattern characterized by the same progression as for forest outlined in eighth notes and Fire—pattern characterized by progression outlined in sixteenth notes) and rename two randomly played phrases (MT1);
- Sight reading of a melody phrase in G-major by singing based on scale-related ascending and descending structures (MT2);
- Improvisation task with the melody in G Major, mostly based on a main triad structure (4 bars, 2/4, piano recording) in which the subject was asked to continue by singing without length restrictions (MT3);
- Play or sing their favorite music piece, which they were instructed to rehearse beforehand (MT4).

All RTs and MTs were recorded as audio tracks and saved without the participant’s names, but in a coded study ID. To assess the accuracy and quality of the music performance, the recorded tasks were independently assessed by three raters, who were all music experts. The raters listened to the performances using high-quality headphones at a fixed volume and were blind to the subject. They were introduced to the checklist assessment, which they used to evaluate the participants’ performances.

**Figure 1:**
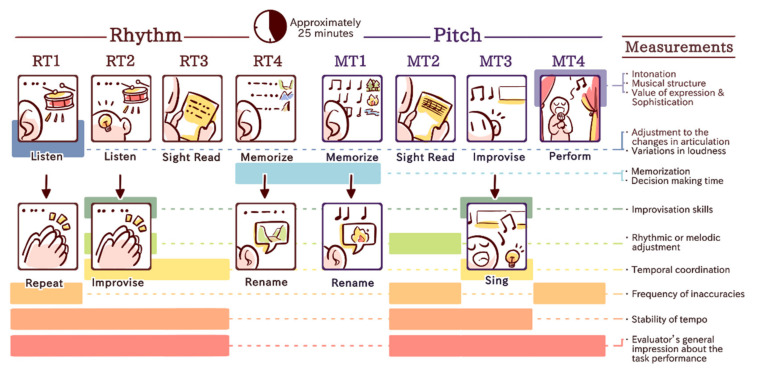
Specific criteria and measurements of the Musical Performance Assessment Scale (MuPAS).

In total, 30 specific criteria (see Figure 1) were set out for all 8 tasks, including the evaluator’s general impression of the task performance (RT1, RT2, RT3, MT2, MT3 and MT4), the frequency of inaccuracies (RT1, MT2 and MT4), the stability of tempo (RT1, RT2, RT3, MT2 and MT3), adjustment to the changes in articulation (RT1), variations in loudness (RT1), rhythmic or melodic adjustment (RT2 and MT2), temporal coordination (RT2, RT3 and MT3), improvisation skills (RT2 and MT3), memorization (RT4 and MT1) and decision-making time (RT4 and MT1), intonation (MT4), musical structure (MT4), value of expression and sophistication (MT4). All the involved music experts evaluated recordings separately using a bipolar scaling method (Likert scale). The rating scale ranged from 0 “min” to 10 “max” for each single task (the maximum possible value was 30 points from all 3 raters; for descriptions, see Table 2). Principal component analysis was applied to assess the internal relationships between the variables and to reduce the variables of the MuPAS to meaningful dimensions. The internal consistency of the musical performance measures was tested in a larger sample to meet statistical requirements for performing a PCA, which demands 5 to 10 participants per variable [100]. Based on the findings, we devised unit-weighted composite scores for six factors: musical expression, rhythmic reproduction, rhythmic improvisation, pitch reproduction, pitch improvisation and rhythmic and pitch memorization. A detailed description of the principal component analysis, the participants and findings are included in the Supplementary Materials for further illustration (see Figure S1 and Table S1). To assess interrater reliability, we ran a correlational analysis based on previous investigations [10,14]. The results indicated high interrater reliability. The findings are shown in the Supplementary Materials (see Tables S2–S7).

### 2.4. Neurophysiological Measurement: Magnetencephalography (MEG)

#### 2.4.1. Stimuli

Auditory-evoked fields (AEFs) were recorded using a Neuromag-122 whole-head MEG system in response to seven different sampled instrumental sounds tones (piano, guitar, flute, bass clarinet, trumpet, violin and drums) and four artificial harmonic complex tones, as performed in previous studies [35,37,51,53]. This set of stimuli is known to evoke the primary auditory P1 response occurring about 50–100 ms after tone onset. It is followed by the N1 complex that peaks around 110–180 ms after tone onset.

#### 2.4.2. Procedure

The AEFs were recorded with a bandpass filter of 0.00 (DC)–330 Hz and a sampling rate of 1000 Hz. The head position inside the Dewar was determined, and the loudness of the stimulation was adjusted to 70 dB SPL, as determined by a Brüel and Kjaer artificial ear (type 4152). Stimuli were presented binaurally without any tasks. Subjects were instructed to listen to the sounds in a relaxed state while watching a silent movie to control their vigilance. In order to obtain a larger signal-to-noise ratio, the sound material was presented for 20 min in a continuous sequence (total of N = 1200 acoustic stimuli; tone length 500 ms each and interstimulus interval randomized between 400–500 ms), resulting in a minimalized influence of superimposed oscillation patterns and enabling robust source modeling as a basis for the additional analysis of the time course, latencies and amplitudes of the auditory-evoked fields. Data analysis was conducted with the BESA Research 6.0 software (MEGIS Software GmbH, Graefelfing, Germany).

#### 2.4.3. Pre-Processing

Prior to averaging, data were inspected to automatically exclude external artifacts using the BESA Research event-related fields (ERF) module. By applying the automatic artifact Scan tool across all participants, on average, 3–7 noisy (bad) channels were excluded, and around 10% of all epochs exceeding a gradient of 600 fT/cm s and amplitudes either exceeding 3000 fT/cm, or falling below 100 fT/cm, were rejected from further analysis. Thereby, a major portion of endogenous artifacts, such as eye blinks, eye movements, cardiac activity, face movements and muscle tensions could be accounted for. A baseline amplitude calculated over the 100 ms interval before the onset of the tones was subtracted from the signals. The responses of each subject were first collapsed into a grand average (about 1000 artifact-free epochs after the rejection of 10% of artifacts afflicted or noisy epochs) in a 100 ms prestimulus to 400 ms poststimulus time window. Based on a spherical head model [101,102], spatio-temporal source modeling was performed to separate the primary response complex from the later secondary responses using a two-dipole model, with one equivalent dipole in each hemisphere [35,37,53,103,104].

#### 2.4.4. Variables

The P1 wave is a composite response complex comprising separate peaks of the earlier primary and later secondary auditory activity and shows large inter-individual differences with respect to shape, the number of subpeaks and the timing of peak latencies. Therefore, the fitting intervals were adjusted from peak onset time either toward the saddle point in the case of a two-peak complex or toward the main peak latency in the case of a merged single P1 peak. Due to developmental maturation, the P1 response complex occurs around 30–70 ms after tone onset in adults [35] but after around 40–90 ms in adolescents and after around 60–110 ms in primary school children [51,53,105,106]. Independent of age, the primary P1 response could clearly be separated from the following later secondary N1 response, which typically starts to develop at the age of 8–10 years [53]. In the first step, the primary source activity was modeled based on one regional source in each hemisphere using predefined fitting intervals around the individual response peaks including their half-side lobes. In the second step, the localization of the fitted regional sources was kept fixed, and the dipole orientation was then fitted to the direction with the highest global field power, keeping its main orientation toward the vertex. The high temporal accuracy of the peak latencies is a general advantage of MEG measurements, widely independent of the exact source location in the auditory cortex [37]. Following previous studies, the right and left P1 and N1 peak latencies were calculated [51,53]. We devised a composite score for the left and right P1 and N1 latencies, namely P1 latency right and left (mean), and N1 latency right and left (mean). Here, “mean” means the average across hemispheres. In addition, an indirect measure of functional lateralization, the absolute P1 latency asynchrony |R-L| [P1(Peak)(|right − left|)] and absolute N1 latency asynchrony |R-L| [N1(Peak)(|right − left|)] were considered. In the second step, we correlated the four MEG variables with the musical performance measurement.

### 2.5. Statistical Analysis

The statistical analysis was divided into three main parts. In the first step, we performed a MANOVA to clarify if there was a significant effect of the diagnoses on the musical performance assessment. For this, we used the six unit-weighted composite scores of the musical performances, musical expression, rhythmic reproduction, rhythmic improvisation, pitch reproduction, pitch improvisation and rhythmic and pitch memorization as dependent variables and the diagnoses (ADD, ADHD, dyslexia and control groups) as the grouping variable. In general, a significant MANOVA could be followed by using discriminant analysis or by separate univariate ANOVAs. Discriminant analysis has the benefit that no corrections for multiple comparisons have to be applied, which is why this approach should be preferred. For completeness, statisticians recommend running both ANOVAs and discriminant analyses as follow-ups for significant MANOVAs [107,108]. Therefore, we ran both discriminant and separate univariate ANOVAs. Discriminant analysis was used in order to illustrate which musical performance variables divided our diagnosis groups best, and separate univariate ANOVAs were used to present mean differences for each of the six main musical performances. As there were unequal group sizes, we run Welch-ANOVAs followed by Games–Howell post hoc analyses for pairwise group comparisons, which is a very robust method [107]. Since we interpreted the results of the MANOVA and discriminant, we did not apply a Bonferroni correction for multi comparisons on the separate univariate ANOVAs but included them for transparency reasons.

In the second step, we used the same procedure for the MEG variables, where we performed a MANOVA, followed by separate univariate ANOVAs and discriminant analysis. The dependent variables were the four composite scores, P1 latency right and left (mean), N1 latency right and left (mean), the absolute P1 latency asynchrony |R-L| and the absolute N1 latency asynchrony |R-L| and the diagnoses (ADD, ADHD, dyslexia and control groups) as the grouping variable.

This approach aimed to select the music performance and MEG variables which discriminated our groups best. In addition, we wanted to uncover whether the music and MEG variables that discriminated our groups best were correlated with each other.

In the third step, we took the music performance and the MEG variables which differentiated our groups best based on both the music performance and MEG discriminant analyses and correlated the remaining few variables. This aimed to determine whether there was an association between the music performance and MEG variables. Afterwards, the correlations were corrected for multiple testing by applying a Benjamini–Hochberg correction.

## 3. Results

### 3.1. Descriptives Statistics of the Musical Performance Variables

Table 2 illustrates the means and standard errors of the musical performance variables of all participants. The means of the individual groups (controls, ADHD, ADD and dyslexics) for the variables under consideration are provided in Section 3 (Table S8) in the Supplementary Materials.

### 3.2. Group Differences in Musical Performance

#### 3.2.1. MANOVA: Mean Differences of Musical Performance

First, we performed a MANOVA to assess whether our six dependent variables, musical expression, rhythmic improvisation, rhythmic reproduction, pitch reproduction, pitch improvisation and rhythmic and pitch memorization, differed in their mean values based on the diagnoses as the grouping variable. Using Pillai’s trace, there was a significant effect of musical performance assessment and diagnosis V = 0.646, *F*(18, 267) = 4.07, *p* < 0.001. Since the MANOVA was significant, we performed separate ANOVAs for the six main criteria of the Musical Performance Assessment.

#### 3.2.2. ANOVAs and Post Hoc Comparison of Musical Performance

In order to test for differences in musical performance between diagnostic groups, we also ran separate one-way ANOVAs followed by post hoc analyses for pairwise group comparisons. As there were unequal group sizes, we ran Welch-ANOVAs followed by Games–Howell post hoc analyses for pairwise group comparisons. All ANOVAs were significant except for the dependent variable rhythmic memorization, as shown in Table 3.

**Figure 2:**
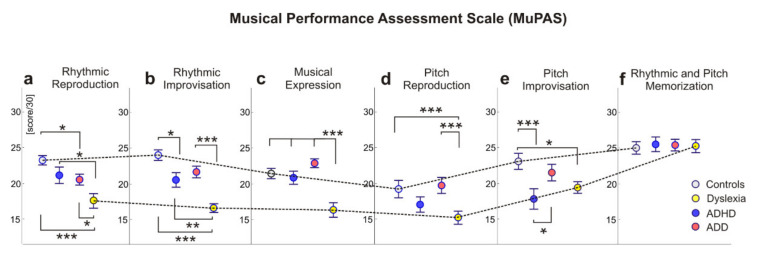
Means and post hoc comparisons of the Musical Performance Assessment Scale by diagnoses. Asterisks indicate the significance (* *p* < 0.05, ** *p* < 0.01, *** *p* < 0.001).

The tables and precise values of the post hoc comparisons of the variables of musical performance are contained in the Supplementary Materials (see Table S8) and are summarized in Figure 2 below. The findings illustrate the mean value differences of the six music performance measures between the diagnoses groups with ADHD, ADD and dyslexia and the control groups. The results revealed that the control group, the ADHD group and the ADD group performed better than the dyslexic group in the rhythmic reproduction, rhythmic improvisation and musical expression tasks. In rhythmic reproduction, the controls scored higher than the ADD group, and in rhythmic and pitch improvisation, the controls performed significantly better than the ADHD group. Adolescents with ADD and controls scored higher in pitch reproduction than the dyslexic group. Only in pitch improvisation did the ADD group outperform the ADHD group, while only the controls scored higher in pitch improvisation than the dyslexic group.

#### 3.2.3. Discriminant Function of Musical Performance

The MANOVA was followed by a discriminant analysis for the variables of the Musical Performance Assessment Scale, which revealed three discriminant functions (see Table S9). The first explained 79.6% of the variance, canonical R^2^ = 0.45, whereas the second explained 13.4%, canonical R^2^ = 0.12 and the third 7%, canonical R^2^ = 0.07. In combination, these discriminant functions significantly discriminated the groups, Λ = 0.45, χ^2^(18) = 72.73, *p* < 0.001, but removing the first function indicated that the second function did not significantly differentiate the four groups Λ = 0.82, χ^2^(10) = 18.2, *p =* 0.052, and the third function also did not significantly differentiate the four groups Λ = 0.93, χ^2^(4) = 6.4, *p =* 0.17. The correlations between the outcomes and the discriminant functions revealed loads onto the first function for the rhythmic improvisation (r = 0.72) and musical expression (r = 0.67). The correlations between the outcomes and the discriminate functions showed loads onto the second non-significant function for pitch improvisation (r = 0.83). The correlations between the outcomes and the discriminate functions showed loads onto the third non-significant function for rhythmic reproduction (r = −0.73).

**Figure 3:**
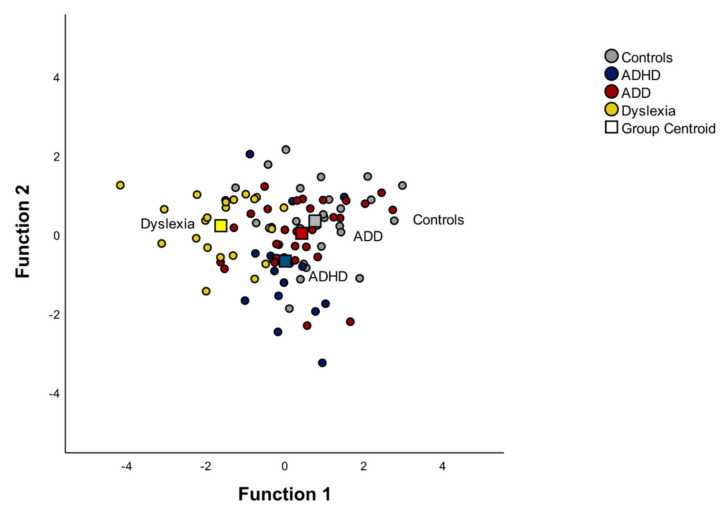
Discriminant function of the Musical Performance Assessment Scale. Function 1 discriminates the dyslexic group from the control, ADD and ADHD groups. The correlations between the outcomes and the discriminant functions revealed that the loads onto the first function are high for the rhythmic improvisation (r = 0.72) and musical expression (r = 0.67).

Therefore, if an arbitrary cut-off of 0.50 is used to decide which of the standardized discriminant coefficients are large, rhythmic improvisation and musical expression discriminate the groups best. The discriminant plot revealed that the first function separated the dyslexic group from the other three groups quite well (see Figure 3). Table S9 in the Supplementary Materials shows all the correlations between the outcomes and the discriminant functions in more detail. In consideration of the results, the discriminant analysis revealed that the rhythmic improvisation and musical expression task variables discriminate the groups best, which is why we discuss them in more detail in this paper.

### 3.3. Descriptives Statistics of the Auditory-Evoked Field Variables

Table 4 illustrates the means and standard errors of the MEG variables of all participants. The means of the individual groups (controls, ADHD, ADD and dyslexics) for the MEG variables under consideration are provided in Section 3 (Table S10) in the Supplementary Materials.

### 3.4. Group Differences in Auditory-Evoked Fields

#### 3.4.1. MANOVA: Mean Differences of Auditory-Evoked Fields

First, we performed a MANOVA to assess whether our four dependent MEG variables, P1 latency right and left, absolute P1 latency asynchrony |R-L|, N1 latency right and left and absolute P1 latency differ in their mean values based on the diagnoses as the grouping variable. Using Pillai’s trace, there was a significant effect of musical performance assessment and diagnosis V = 0.420, F(12, 267) = 3.62, *p* < 0.001. Since the MANOVA was significant, we performed separate ANOVAs for the four MEG variables.

#### 3.4.2. ANOVAs and Post Hoc Comparisons of Auditory-Evoked Fields

In order to test for differences in the auditory-evoked fields between the disorder groups, we also ran separate one-way ANOVAs followed by post hoc analyses for pairwise group comparisons. As there were unequal group sizes, we run Welch-ANOVAs followed by Games–Howell post hoc analyses for pairwise group comparisons. All ANOVAs were significant except for the dependent variable absolute N1 latency asynchrony |R-L|, as shown in Table 5.

The tables and precise values of the post hoc comparisons of the MEG variables are shown in the Supplementary Materials (see Table S10). The findings illustrate the mean value differences of the four MEG measures between the disorder groups ADHD, ADD and dyslexia and the controls. The results revealed that the dyslexic group had significantly later P1 latencies right and left (mean) than the control and the ADHD group. Additionally, the control group showed significantly lower absolute P1 latency asynchrony |R-L| than all disorder groups. The control group and the ADHD also demonstrated earlier N1 latency right and left (mean) than the dyslexic group. In addition, the control group showed earlier N1 latency right and left (mean) than the ADD group.

#### 3.4.3. Discriminant Function of Auditory-Evoked Fields

The MANOVA was followed by a discriminant analysis for the MEG variables, which revealed three discriminant functions (see Table S11). The first explained 71.8% of the variance, canonical R^2^ = 0.28, whereas the second explained 26.8%, canonical R^2^= 0.13 and the third 1.4%, canonical R^2^ = 0.007. In combination, these discriminant functions significantly discriminated the groups, Λ = 0.61, χ^2^(12) = 42.62, *p* < 0.001. Removing the first function indicated that the second function also significantly differentiated the four groups Λ = 0.86, χ^2^(10) = 12.94 *p =* 0.044, while the third function did not significantly differentiate the four groups Λ = 0.99, χ^2^(4) = 0.4, *p =* 0.72.

The correlations between the outcomes and the discriminant functions revealed loads onto the first function for the absolute P1 latency asynchrony |R-L| (r = 0.97). The correlations between the outcomes and the discriminate functions showed loads onto the second function for P1 latency right and left (mean) (r = 0.82) and for N1 latency right and left (mean) (r = 0.68). The correlations between the outcomes and the discriminate functions showed loads onto the third non-significant function for absolute N1 latency asynchrony |R-L|(r = −0.28).

**Figure 4:**
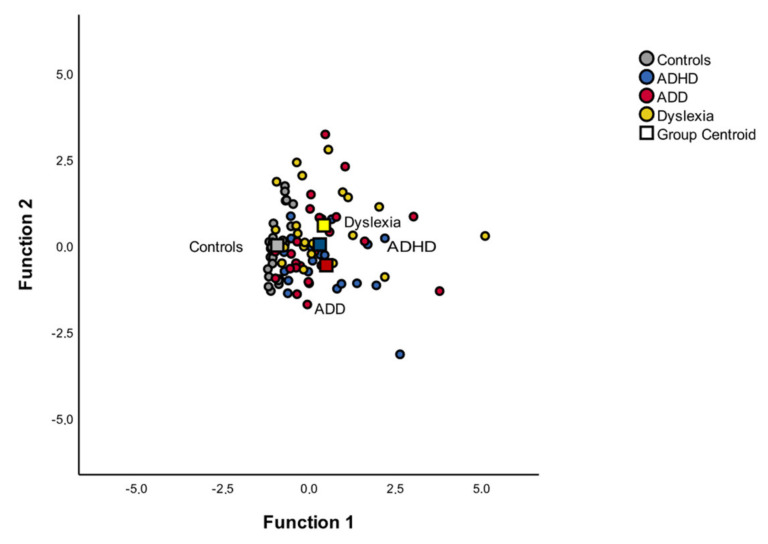
Discriminant function of the MEG variables. Function 1 discriminates the controls from the dyslexic, ADD and ADHD groups, while the second function discriminates the dyslexic group from the control, ADD and ADHD groups. The correlations between the outcomes and the discriminant functions revealed that the loads onto the first function are high for the absolute P1 latency asynchrony |R-L| (r = 0.97), while the correlations between the outcomes and the discriminant functions revealed that the loads onto the second function are high for P1 latency right and left (mean) (r = 0.82) and for N1 latency right and left (mean) (r = 0.68).

The absolute P1 latency asynchrony |R-L|, P1 latency right and left (mean) and the N1 latency right and left (mean) are above the recommended arbitrary cut off of 0.50. The discriminant plot revealed that the first function clearly separated the control group from the three disorder groups (see Figure 4), whereas the second function separated the control, ADHD and ADD groups from the dyslexic group.

### 3.5. Correlations of Musical Performance and MEG

**Figure 5:**
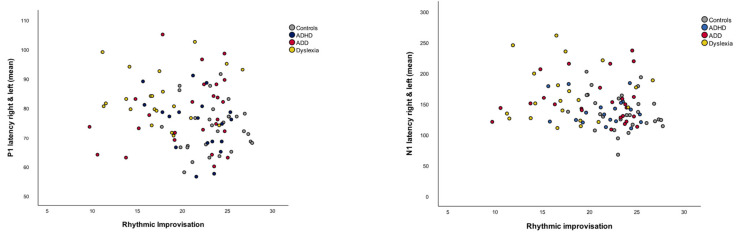
Correlation plots of rhythmic improvisation with P1 latency right and left (mean) and N1 latency right and left (mean). Better rhythmic improvisation is associated with early P1 and N1 latencies (mean). Both correlations remain significant after Benjamini–Hochberg correction for multiple testing (*p* < 0.05).

After group comparisons, we performed correlational analyses in order to provide information about the relationship between the musical performance measures and the neurophysiological variables. We therefore used the two music performance variables which discriminated our groups best. These were the rhythmic improvisation and musical expression, which were correlated with the MEG variables (for descriptions, see Table 4). While we could not detect a relationship between P1 and N1 responses and musical expression, rhythmic improvisation correlated with two of the MEG variables under consideration (see Table 6 and Figure 5).

## 4. Discussion

The considerable worldwide prevalence of ADHD, ADD and dyslexia (5–10%) and the known benefits of musical training on neuronal processing and behavior [51,53] highlight the importance of gaining a better insight into and understanding of auditory processing in order to optimize musical education and to develop new pedagogic interventions for children/adolescents with developmental and learning disorders. Therefore, this study aimed to evaluate possible characteristic differences in music performance and auditory-evoked field variables in adolescents with dyslexia, ADHD and ADD. In addition, we sought to uncover potential correlations between musical performance and MEG response patterns. Since our previous study focused on music perception and linked atypical neurofunctional patterns to individual differences in music perception [51], we now aimed to go beyond music perception and address musical capacities from the perspective of musical performance and used musical performance assessment measurements based on already established test designs [99] and analysis procedures of previous research [14]. Through this, we could show that, compared to controls, dyslexic children/adolescents score lower in basic music-hearing tasks (frequency and onset ramp discrimination) and complex sound-processing tasks (meter, rhythm, and melody differentiation), and that children/adolescents with ADHD score lower in complex rhythmic and melodic perception tasks [51]. In contrast, children/adolescents with ADD did not show any auditory impairments at all [51].

In our current study, musical performance differed significantly across groups. In general, the control, ADD and ADHD groups scored higher than the dyslexic participants in almost all measures of musical performance, except for the rhythmic and pitch memorization task, in which all groups scored similarly. Since rhythmic and pitch memorization are based on memorizing melodic and rhythmic phrases, it could be assumed that these measures reflect not only musical performance mechanisms but also require short-term memory ability. The reason why we could not detect mean differences could be attributed to the fact that tasks were not long enough in order to uncover individual differences.

For the interpretation of our results, we mainly relied on discriminant analysis, which provided information about which of our variables separate our participants. The discriminant analysis of the musical performance measures revealed that rhythmic improvisation and musical expression discriminated the groups best. In the following, we discuss these variables and the underlying concepts in more detail. We assumed that compared to the control and ADD groups, the dyslexic participants would perform worse in rhythmic improvisation and musical expression. However, we did not expect that the ADHD group would perform better than the dyslexic group, since in previous studies, we noted music perception deficits in both the ADHD and the dyslexic participants. Even though rhythm-related and musical perception deficits have been reported in individuals with ADHD [51,81,84], in our current study, adolescents with ADHD and ADD scored similarly to controls in rhythmic improvisation and musical expression. Subsequently, as results in music performance may differ from results in music perception, research findings in music performance should not be transferred to music perception and vice versa.

In former investigations, we noted that individuals with dyslexia suffer from severe auditory deficits compared to children/adolescents with ADHD, ADD and controls [51]. In our current study, individuals with dyslexia performed worse in rhythmic improvisation than adolescents with ADHD and ADD and the controls. The ability to encode incoming temporal information is not only crucial for musical but also for phonological processing. Goswami [67] postulates that auditory rhythmic entrainment is impaired if individuals have specific difficulties with Theta and Delta oscillators. This auditory entrainment not only affects attentional but also auditory integration. The phonological impairments of individuals with dyslexia can therefore be understood as auditory sensory impairments. This supports assumptions of language disorder frameworks such as the temporal sampling framework (TSF) [67] and the PRISM [47], which suggest that timing difficulties of individuals with dyslexia may be caused by auditory sensory integration impairment of incoming acoustic signals. Since the PRISM is based on shared mechanisms of language and speech, it is also applicable to musical performance. In the light of the present findings, it is plausible to assume that the rhythmic impairment of individuals diagnosed with dyslexia affects the musical and language domain in a similar way.

An indirect aspect, namely creativity, could be a further reason why subjects with ADHD and ADD perform better than adolescents with dyslexia. Musical improvisation and expression are defined by the ability to perform music in a creative and spontaneous way [109]. As ADHD symptoms are associated with more flexible association networks [110] and better creative performance [111,112,113,114], one could postulate that both aspects combined could serve as an explanation as to why adolescents with ADHD and ADD score higher in rhythmic improvisation and musical expression than dyslexics.

In contrast, dyslexics have not been found to be more creative or show greater variability in creativity than peers without dyslexia [115]. It is known that due to a variety of basic auditory deficits [68,69,116], dyslexics show impaired development of language abilities such as the acquisition of phonological representations, literacy skills [53,62,117] and the perception of metrical structure in music [65]. Additionally, dyslexics are impaired in controlling brief temporal components of acoustic spectra in their motor output [118,119] and in anticipating and maintaining the beat in rhythmic entrainment tasks [120,121]. These temporal impairments may lead to the abovementioned difficulties in musical and rhythmical perception and production. The discriminant analysis of auditory-evoked fields revealed that the first function distinguished the control group from the disorder groups based on P1 latency asynchrony |R-L|, which is in line with previous research [51,53]. The second function of P1 and N1 latencies (mean) distinguished the control, ADD and ADHD groups from the dyslexic group. We then, correlated the music performance variables rhythmic improvisation and musical expression, which discriminated the dyslexic group from all other groups, with all MEG variables. Correlational analyses on musical performance and auditory-evoked fields revealed a relationship between rhythmic improvisation and P1 and N1 latencies (mean). The primary P1 component is thought to be a marker for musical talent [53,104] and can already be measured in early childhood [122]. The N1 response usually emerges later, at about 8–10 years of age [106,122]. The N1 component, which reflects sensory stimuli processing, is linked to attention-specific processes [123] and shows a strong context dependency and learning-induced plasticity [37]. The later N1 latency (mean) and the weaker rhythmic improvisation performance of adolescents with dyslexia not only could be understood as a perceptual impairment, but also as a deficit in sensorimotor motor translations, which makes individuals insensitive to accurately reproducing musical input. Hence, it is crucial to consider that sensory processing influences efficiency in motor output [82].

As a sign of natural development and maturity, the latencies of the primary P1 and secondary N1 response component accelerate up to the age of 15 years [105,122,124]. ADHD, ADD and dyslexia are characterized by specific neuroanatomical and neurofunctional differences in the auditory cortex. The MEG source waveforms of children/adolescents with ADD are known to be shifted in latency but balanced in shape, while the response patterns of children/adolescents with ADHD were temporally expanded in the left and diminished in the right hemisphere, and in the dyslexic group, the P1 peak was enlarged. Further, all disorder groups showed a higher P1 latency asynchrony |R-L| [51,53]. The P1 latency asynchrony |R-L|, which indicates a shift in latency, differentiated the control group from all disorder groups best in this investigation. This asynchrony corresponds to a reduced integration of left hemispheric fine-grained and right hemispheric supra-segmental signal representations, which lead to difficulties in discriminating onsets of syllables and perceiving rhythmic structures in speech and music. These difficulties are characteristic for children with dyslexia [116,125] and are frequently associated with AD(H)D [126]. There is evidence that children and adolescences with AD(H)D demonstrate an atypical development of the N1 component with growing latency over time, whereas non-affected individuals are characterized by a declining latency [127]. It seems possible that by means of attentional training, adults with AD(H)D develop compensatory mechanisms as a part of maturity and cognitive enhancement [128,129]. Compared with normal average readers, dyslexic children exhibit prolonged latencies of auditory-evoked potentials, possibly reflecting disturbances in written language acquisition [130,131]. The negative correlation between the rhythmic improvisation and P1 and N1 latencies in our study implies that the earlier the P1 and N1 latencies (mean), the better the rhythmic improvisation.

Neurophysiological studies in musicians have shown brain plasticity induced by musical training, such as enhanced activation in the auditory cortex [132,133,134], more pronounced structural and functional connectivity [34,135,136,137,138] and intracerebral synchronization [139,140]. Musical training is known to positively affect the accuracy of auditory perception [53,141,142,143,144,145], language development [146,147,148,149,150,151,152,153] and motor functions [38,154,155]. There are strong links between rhythmic and linguistic abilities [156,157,158,159,160,161,162,163]. Additionally, making music is associated with beneficial influences in general cognitive and executive functions such as planning, self-control, working memory [164,165,166] and the conscious control of attention [167,168]. In particular, frequent musical performance rehearsals optimize and strengthen neuronal interconnection by changing the timing and synchronization as well as the number and strength of stimulating and inhibiting synaptic connections and postsynaptic potentials [34,169,170,171,172,173,174,175,176,177,178,179].

Patients with ADHD and ADD benefit from music therapy using improvisational musical input, as it has been shown to improve emotional lability, psychosomatic symptoms and attention [180,181,182]. The additional advantages of music-based training programs are their motivating, playful approach, the possibility of speech-free interaction and the use of resources such as the joy of movement, creativity and openness [183,184] that often characterize children with ADHD [185]. Dyslexic children could benefit from music and especially rhythmic training, leading to improved brain circuitry for music and language processes. In addition, the temporal and rhythmical features of music could positively affect temporal processing deficits [64,186,187].

Further studies should be based on larger numbers of participants and focus on the role of the P1 and N1 latencies in ADHD, ADD and dyslexia and how they can be influenced by specific musical training. Research outlined that the synchronization and balancing of right and left auditory responses increases musical practice in controls and adolescents with dyslexia, ADHD and ADD [51,53]. As balanced and reduced latencies are correlated with more efficient and enhanced auditory processing and attention and literacy skills, it can be assumed that the shorter the latency, the faster and more precise the auditory processing [51,53]. In this investigation, we could also detect that P1 and N1 latencies showed a negative correlation to rhythmic improvisation in music performance, which suggests that enhanced auditory processing can probably also predict individual differences in sensorimotor timing ability. In addition, future studies should shed light on how P1 and N1 latencies can be reduced by instructional musical input, which would enrich therapeutic methods for individuals with ADHD, ADD and dyslexia.

In conclusion, our data provide novel insight into differences of music processing and performance in adolescents with and without neurodevelopmental disorders. A better understanding of these distinct differences in musical performance and underlying neurobiological factors may help to develop tailored preventions or interventions for individuals with ADHD, ADD and dyslexia. These should include sensory–motor training and the training of fine-grained auditory skills such as pitch, timing and timbre perception tasks.

## Figures and Tables

**Table 1:** Description of participants.

Parameters	Categories	Controls	ADHD	ADD	Dyslexic
Number of subjects		28	19	28	21
Age in years	mean ± SD	14.48 ± 1.12	14.05 ± 1.43	14.32 ± 1.78	13.64 ± 1.17
Musical Status *	mean	9.73 ± 6.03	5.94 ± 6.55	7.11 ± 9.02	5.74 ± 7.18
Sex	female	14	2	8	10
	male	14	17	20	11
Handedness	right	24	16	22	16
	left	4	3	6	5

* Musical status = product of the number of years of formal music education and the number of hours per week spent practicing an instrument or singing. To give an example, a musical status of 6 could be defined by 6 years of formal music education and 1 h spent practicing.

**Table 2:** Descriptive statistics of the six variables of the Musical Performance Assessment Scale.

Variables	Mean (M)	Standard Error (SE)
Rhythmic Reproduction (corr/30)	20.67	0.45
Rhythmic Improvisation (corr/30)	20.93	0.47
Musical Expression (corr/30)	20.66	0.44
Pitch Reproduction (corr/30)	18.03	0.52
Pitch Improvisation (corr/30)	20.71	0.53
Rhythmic and Pitch Memorization (corr/30)	25.51	0.32

Level of performance: excellent/very good (30–24); good/almost good (23–18); satisfactory/almost satisfactory (17–12); unsatisfactory (11–3).

**Table 3:** ANOVA results of the six different variables of the Musical Performance Assessment Scale.

Variables	F	p	ω
Rhythmic Reproduction	(3, 94) = 8.95	<0.001	0.45
Rhythmic Improvisation	(3, 94) = 12.77	<0.001	0.52
Musical Expression	(3, 94) = 13.09	<0.001	0.53
Pitch Reproduction	(3, 94) = 6.02	<0.001	0.38
Pitch Improvisation	(3, 94) = 4.93	=0.003	0.35
Rhythmic and Pitch Memorization	(3, 94) = 0.25	=0.862	--

**Table 4:** Descriptive statistics of the four MEG variables.

Variables	Mean (*M*)	Standard Error (*SE*)
P1 latency right and left (mean)	77.26	1.08
absolute P1 latency asynchrony |R-L|	8.19	0.91
N1 latency right and left (mean)	147.99	3.84
absolute N1 latency asynchrony |R-L|	24.89	2.62

**Table 5:** ANOVA results of the four MEG variables.

Variables	F	p	ω
P1 latency right and left (mean)	(3, 90) = 5.06	=0.003	0.34
absolute P1 latency asynchrony |R-L|	(3, 90) = 11.55	<0.001	0.50
N1 latency right and left (mean)	(3, 90) = 3.64	=0.016	0.28
absolute N1 latency asynchrony |R-L|	(3, 90) = 0.39	=0.764	--

**Table 6:** Correlations of MEG variables under consideration with the musical performance variables rhythmic improvisation and musical expression.

	P1 Latency Right and Left (mean)	Absolute P1 Latency Asynchrony |R-L|	N1 Latency Right and Left (mean)	Absolute N1 Latency Asynchrony |R-L|
Rhythmic Improvisation	−0.290 **	−0.184	−0.298 **	−0.189
Musical Expression	−0.137	−0.133	−0.135	−0.147

** *p* < 0.001 (uncorrected, two-tailed).

## Data Availability

Data are contained within the article or Supplementary Materials.

## Supplementary Materials

The following are available online at https://www.mdpi.com/article/10.3390/brainsci12020127/s1, Figure S1: scree plot of the principal components analysis, Table S1: summary of exploratory factor analysis results, Tables S2–S7: interrater correlations of the six main criteria of the MuPAS, Table S8: post hoc analyses of the six variables of the Musical Performance Assessment Scale, Table S9: correlations of the outcome variables and the discriminant functions, Table S10: post hoc analyses of the MEG variables, Table S11: correlations of the outcome variables and the discriminant functions.
