# Supplementary material for: Musical Performance in Adolescents with ADHD, ADD and Dyslexia—Behavioral and Neurophysiological Aspects
Source: Brain Sci. 2022 Jan 18;12(2):127. doi: 10.3390/brainsci12020127 (PMC8870592; doi:10.3390/brainsci12020127)
Supplement: Supplementary file 1 [file brainsci-12-00127-s001.zip › brainsci-1519830-supplementary.pdf]

1. Principal Component Analysis (PCA)

To illustrate the internal relationships of our measures, we performed a factor analysis with the aim to assess the characteristics of our rating criteria. We therefore used a larger sample of participants (N = 185) who did not differ in musical practice between the ages 8 and 18 to make factor analysis robust.

A principal component analysis (PCA) was conducted on the 27 musical performance rating criteria with orthogonal rotation (varimax). The Kaiser–Meyer–Olkin measure verified the sampling adequacy for the analysis, KMO 0.91, and all individual KMO variables were >0.58, which is well above the acceptable limit of 0.5. Bartlett's test of sphericity χ^2 (351) = 9805.67. $p < 0.001$ indicated that correlations between items were sufficiently large for PCA. An initial analysis was run to obtain eigenvalues for each component in the data. Six components had eigenvalues over 1 and in combination explained 89.54 percent of the variance. The convergence of the scree plot (see Figure S1) as well as the large number of participants indicated that six criteria should be used. Component one represents musical expression, component two rhythmic improvisation, component three rhythmic reproduction, component four pitch reproduction, component five pitch improvisation and component six rhythmic and pitch memorization. Table S1 below shows the factor loadings after rotation.

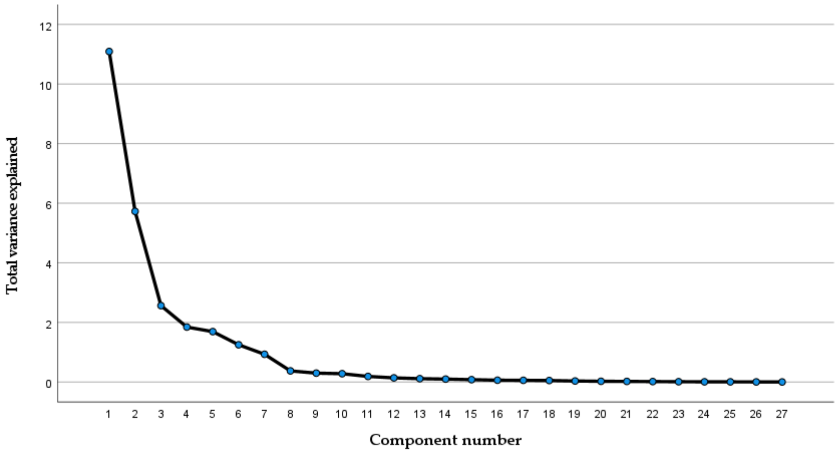

Figure S1. Scree plot of the principal component analysis.

Table S1. Summary of exploratory factor analysis results (N = 185).

	Rotated Factor Loadings					
**Item**	**Musical Expression**	**Rhythmic Improvisation**	**Rhythmic Reproduction**	**Pitch Inprovisation**	**Pitch Reproduction**	**Rhythm and Pitch Memorization**
RT1 general impression		0.39	**0.85**			
RT1 frequency of inaccuracies		0.37	**0.86**			
RT1 stability of tempo		0.39	**0.84**			
RT1 changes in articulation		0.36	**0.86**			
RT1 variations in loudness		0.33	**0.87**			
RT2 general impression		**0.88**	0.37			
RT2 rhythmic adjustment		**0.87**				
RT2 stabilty of tempo		**0.86**	0.35			
RT2 temporal coordination		**0.87**	0.35			

RT2 improvisation skills		**0.88**	0.35			
RT4 memorization						**0.86**
RT4 decision-making time						**0.84**
MT1 memorization						**0.86**
MT1 decision-making time						0.34
MT2 general impression	0.33				**0.88**	
MT2 frequency of inaccuracies	0.31				**0.87**	
MT2 stability of tempo	0.32				**0.87**	
MT2 melodic adjustment					**0.84**	
MT3 general impression				**0.91**		
MT3 stability of tempo				**0.93**		
MT3 temporal coordination				**0.92**		
MT3 improvisation skills				**0.91**		
MT4 general impression	**0.95**					
MT4 frequency of inaccuracies	**0.96**					
MT4 intonation	**0.96**					
MT4 musical structure	**0.94**					
MT4 value of musical expression and sophistication	**0.95**					
Total variance explained	11.09	5.73	2.57	1.85	1.69	1.25
% of variance	18.99	17.28	16.38	14.52	13.11	9.26
Cronbach's α	0.997	0.978	0.978	0.991	0.969	0.746

* Note that we also coined an equivalent rhythmic concept for RT3 (specific criteria: general impression, stability of tempo, temporal coordination) which failed to be consistent and therefore was excluded from the final analysis. Values below 0.3 were not provided. The Cronbach's α is based on the values in bold. The values in bold of each factor were used to determine unit-weighted composite scores which resulted in the six main scores (musical expression, rhythmic reproduction, rhythmic improvisation, pitch reproduction, pitch improvisation and rhythmic and pitch memorization).

2. Interrater Reliability

We also assessed the interrater reliability of our ratings for the 96 participants of our current study. Therefore, we ran correlational analysis as illustrated in Tables S2–S7. The results revealed that the ratings showed high interrater agreement.

Table S2. Interrater correlations of the variable rhythmic reproduction.

Rhythmic Reproduction	**Rater 1**	**Rater 2**	**Rater 3**
Rater 1		0.683 **	0.739 **
Rater 2			0.642 **
Rater 3			

** $p < 0.001$ (uncorrected, two-tailed).

Table S3. Interrater correlations of the variable rhythmic improvisation.

Rhythmic Improvisation	**Rater 1**	**Rater 2**	**Rater 3**
Rater 1		0.698 **	0.784 **
Rater 2			0.703 **
Rater 3			

** $p < 0.001$ (uncorrected, two-tailed).

Table S4. Interrater correlations of the variable pitch reproduction.

Pitch Reproduction	**Rater 1**	**Rater 2**	**Rater 3**
Rater 1		0.938 **	0.891 **
Rater 2			0.914 **
Rater 3			

** $p < 0.001$ (uncorrected, two-tailed).

Table S5. Interrater correlations of the variable pitch improvisation.

Pitch Improvisation	**Rater 1**	**Rater 2**	**Rater 3**
Rater 1		0.895 **	0.822 **
Rater 2			0.858 **
Rater 3			

** $p < 0.001$ (uncorrected, two-tailed).

Table S6. Interrater correlations of the variable musical expression.

Musical Expression	**Rater 1**	**Rater 2**	**Rater 3**
Rater 1		0.901 **	0.928 **
Rater 2			0.924 **
Rater 3			

** $p < 0.001$ (uncorrected, two-tailed).

Table S7. Interrater correlations of the variable rhythmic and pitch memorization.

Rhythmic and Pitch Memorization	**Rater 1**	**Rater 2**	**Rater 3**
Rater 1		0.444 **	0.551 **
Rater 2			0.816 **
Rater 3			

** $p < 0.001$ (uncorrected, two-tailed).

3. Behavioral Investigation of Musical Performance Assessment Scale

3.1. Games–Howell Post Hoc *Analyses for the Musical Performance Assessment Scale*

Table S8. Post hoc analyses of the six musical performance variables.

Variables	Group	Means and Standard Error	Post Hoc Comparisons	*t*	df	*p*	*r*
Rhythmic Reproduction	Controls	*M* = 23.05; *SE* = 0.51	controls vs. ADHD	1.6	94	0.113	--
			controls vs. ADD	2.37	94	0.02	0.24
			controls vs. dyslexia	5.15	94	<0.001	0.47
	ADHD	*M* = 21.17; *SE* = 0.69	ADHD vs. ADD	0.53	94	0.599	--
			ADHD vs. dyslexia	3.14	94	0.002	0.31
	ADD	*M* = 20.55; *SE* = 0.93	ADD vs. dyslexia	2.9	94	0.005	0.29
	dyslexia	*M* = 17.27; *SE* = 0.99					
Rhythmic Improvisation	Controls	*M* = 23.36; *SE* = 0.67	controls vs. ADHD	2.18	94	0.032	0.28
			controls vs. ADD	1.42	94	0.159	--
			controls vs. dyslexia	6	94	<0.001	0.53
	ADHD	*M* = 20.80; *SE* = 0.96	ADHD vs. ADD	−0.9	94	0.37	--
			ADHD vs. dyslexia	3.36	91	0.001	0.33
	ADD	*M* = 21.87; *SE* = 0.82	ADD vs. dyslexia	4.63	94	<0.001	0.43
	dyslexia	*M* = 16.62; *SE* = 0.79					
Musical Expression	Controls	*M* = 21.75; *SE* = 0.81	controls vs. ADHD	0.71	94	0.48	--
			controls vs. ADD	−0.84	94	0.403	--
			controls vs. dyslexia	5.11	94	<0.001	0.47
	ADHD	*M* = 20.99; *SE* = 0.76	ADHD vs. ADD	−1.45	94	0.149	--
			ADHD vs. dyslexia	3.95	94	<0.001	0.38
	ADD	*M* = 22.56; *SE* = 0.46	ADD vs. dyslexia	5.86	94	<0.001	0.52
	dyslexia	*M* = 16.50; *SE* = 0.87					
Pitch Reproduction	Controls	*M* = 19.55; *SE* = 1.16	controls vs. ADHD	1.78	94	0.078	--
			controls vs. ADD	−0.16	94	0.87	--
			controls vs. dyslexia	3.56	94	<0.001	0.35
	ADHD	*M* = 17.04; *SE* = 0.81	ADHD vs. ADD	−1.93	94	0.057	--
			ADHD vs. dyslexia	1.55	94	0.125	--
	ADD	*M* = 19.76; *SE* = 0.91	ADD vs. dyslexia	3.72	94	<0.001	0.36
	dyslexia	*M* = 14.74; *SE* = 0.71					
Pitch Improvisation	Controls	*M* = 22.76; *SE* = 0.92	controls vs. ADHD	3.47	94	<0.001	0.34
			controls vs. ADD	0.84	94	0.403	--
			controls vs. dyslexia	2.44	94	0.017	0.25
	ADHD	*M* = 17.77; *SE* = 1.29	ADHD vs. ADD	−2.71	94	0.008	0.27
			ADHD vs. dyslexia	−1.03	94	0.306	--
	ADD	*M* = 21.67; *SE* = 0.99	ADD vs. dyslexia	1.66	94	0.099	--
	dyslexia	*M* = 19.34; *SE* = 0.71					
Rhythmic and Pitch Memorization	Controls	*M* = 25.09; *SE* = 0.66	controls vs. ADHD	−0.67	94	0.507	--
			controls vs. ADD	−0.6	94	0.552	--
			controls vs. dyslexia	−0.75	94	0.455	--
	ADHD	*M* = 25.72; *SE* = 0.69	ADHD vs. ADD	0.13	94	0.891	--
			ADHD vs. dyslexia	−0.05	94	0.961	--
	ADD	*M* = 25.29; *SE* = 0.55	ADD vs. dyslexia	−0.19	94	0.851	--
	dyslexia	*M* = 25.77; *SE* = 0.68					

3.2. *Discriminant Functions for the Musical Performance Assessment Scale*

Table S9. The table shows the correlations of the outcome variables and the discriminant functions of the musical performance variables. We used an arbitrary cutoff of 0.50 to decide which of the variables were large enough to discriminate the groups.

	**Function 1**	**Function 2**	**Function 3**
Rhythmic Improvisation	0.724 *	0.149	−0.330
Musical Expression	0.679 *	−0.294	0.515
Rhythmic and Pitch Memorization	−0.083 *	−0.066	0.075
Pitch Improvisation	0.282	0.786 *	0.437
Rhythmic Reproduction	0.476	0.266	−0.588 *
Pitch Reproduction	0.459	0.154	−0.505 *

* indicates the largest absolute correlation between each variable and any discriminant function.

4. Neurophysiological Investigation

4.1. *Games–Howell* Post Hoc *Analyses for the MEG Variables*

Table S10. Post hoc analyses of the four variables of the auditory-evoked fields.

**Variables**	**Group**	**Means and Standard Error**	**Post Hoc Comparisons**	***t***	**df**	***p***	***r***
P1 latency right and left (mean)	Controls	*M* = 73.41; *SE* = 1.56	controls vs. ADHD	−0.46	90	0.649	--
			controls vs. ADD	−1.76	90	0.081	--
			controls vs. dyslexia	−3.69	90	<0.001	0.36
	ADHD	*M* = 74.74; *SE* = 2.23	ADHD vs. ADD	−1.14	90	0.258	--
			ADHD vs. dyslexia	−2.91	90	0.005	0.29
	ADD	*M* = 78.14; *SE* = 2.37	ADD vs. dyslexia	−1.94	90	0.055	--
	dyslexia	*M* = 83.78; *SE* = 1.97					
absolute P1 latency asynchrony|*R-L*|	Controls	*M* = 1.38; *SE* = 0.23	controls vs. ADHD	−5.09	90	<0.001	0.47
			controls vs. ADD	−4.39	90	<0.001	0.42
			controls vs. dyslexia	−4.23	90	<0.001	0.41
	ADHD	*M* = 12.84; *SE* = 1.99	ADHD vs. ADD	1	90	0.32	--
			ADHD vs. dyslexia	0.92	90	0.36	--
	ADD	*M* = 10.52; *SE* = 1.64	ADD vs. dyslexia	−0.04	90	0.965	--
	dyslexia	*M* = 10.62; *SE* = 2.29					
N1 latency right and left (mean)	Controls	*M* = 135.51; *SE* = 6.45	controls vs. ADHD	−0.26	90	0.793	--
			controls vs. ADD	−2.02	90	0.046	0.21
			controls vs. dyslexia	−2.91	90	0.005	0.29
	ADHD	*M* = 138.29; *SE* = 5.21	ADHD vs. ADD	−1.56	90	0.123	--
			ADHD vs. dyslexia	−2.39	90	0.019	0.24
	ADD	*M* = 155.24; *SE* = 7.26	ADD vs. dyslexia	−0.96	90	0.341	--
	dyslexia	*M* = 165.38; *SE* = 9.83					
absolute N1 latency asynchrony |*R-L*|	Controls	*M* = 21.01; *SE* = 5.06	controls vs. ADHD	−0.46	90	0.649	--
			controls vs. ADD	−0.87	90	0.384	--
			controls vs. dyslexia	−0.94	90	0.348	--
	ADHD	*M* = 24.47; *SE* = 6.34	ADHD vs. ADD	−0.34	90	0.734	--
			ADHD vs. dyslexia	−0.43	90	0.669	--
	ADD	*M* = 27.14; *SE* = 4.19	ADD vs. dyslexia	−0.11	90	0.915	--
	dyslexia	*M* = 27.97; *SE* = 5.86					

4.2. Discriminant Functions for the MEG Variables

Table S11. The table shows the correlations of the outcome variables and the discriminant functions of the MEG variables. We used an arbitrary cutoff of 0.50 to decide which of the variables were large enough to discriminate the groups.

	**Function 1**	**Function 2**	**Function 3**
P1 latency right and left (mean)	0.413	0.823 *	−0.342
absolute P1 latency asynchrony |R-L|	0.979 *	−0.193	0.019
N1 latency right and left (mean)	0.356	0.677 *	−0.639
absolute N1 latency asynchrony |R-L|	0.158	0.127	0.275 *

* indicates the largest absolute correlation between each variable and any discriminant function.
